# Ripeness Prediction of Postharvest Kiwifruit Using a MOS E-Nose Combined with Chemometrics

**DOI:** 10.3390/s19020419

**Published:** 2019-01-21

**Authors:** Dongdong Du, Jun Wang, Bo Wang, Luyi Zhu, Xuezhen Hong

**Affiliations:** 1 College of Biosystems Engineering and Food Science, Zhejiang University, Hangzhou 310058, China; dudd@zju.edu.cn (D.D.); 21613008@zju.edu.cn (B.W.); 3130100405@zju.edu.cn (L.Z.); 2 Key Laboratory of On Site Processing Equipment for Agricultural Products, Ministry of Agriculture and Rural Affairs, Hangzhou 310058, China; xzhong@cjlu.edu.cn; 3 College of Quality & Safety Engineering, China Jiliang University, Hangzhou 310018, China

**Keywords:** electronic nose, nondestructive detection, kiwifruit, ripeness, SSC, firmness

## Abstract

Postharvest kiwifruit continues to ripen for a period until it reaches the optimal “eating ripe” stage. Without damaging the fruit, it is very difficult to identify the ripeness of postharvest kiwifruit by conventional means. In this study, an electronic nose (E-nose) with 10 metal oxide semiconductor (MOS) gas sensors was used to predict the ripeness of postharvest kiwifruit. Three different feature extraction methods (the max/min values, the difference values and the 70th s values) were employed to discriminate kiwifruit at different ripening times by linear discriminant analysis (LDA), and results showed that the 70th s values method had the best performance in discriminating kiwifruit at different ripening stages, obtaining a 100% original accuracy rate and a 99.4% cross-validation accuracy rate. Partial least squares regression (PLSR), support vector machine (SVM) and random forest (RF) were employed to build prediction models for overall ripeness, soluble solids content (SSC) and firmness. The regression results showed that the RF algorithm had the best performance in predicting the ripeness indexes of postharvest kiwifruit compared with PLSR and SVM, which illustrated that the E-nose data had high correlations with overall ripeness (training: R^2^ = 0.9928; testing: R^2^ = 0.9928), SSC (training: R^2^ = 0.9749; testing: R^2^ = 0.9143) and firmness (training: R^2^ = 0.9814; testing: R^2^ = 0.9290). This study demonstrated that E-nose could be a comprehensive approach to predict the ripeness of postharvest kiwifruit through aroma volatiles.

## 1. Introduction

Kiwifruit is one of the most valuable fruits because it is native to China and popular around the world. Commercially, it is usually harvested when it is considered mature, but not yet edible for consumers. The postharvest kiwifruit continues to ripen for a period until it is at the optimal “eating ripe” stage when the taste and flavor are the best, although soon thereafter it becomes overripe and unacceptable. It is very difficult to identify the ripeness of postharvest kiwifruit during ripening just by the external features, such as size, shape and color. Previous studies conducted on consumer acceptance have showed that the eating ripeness of kiwifruit is essentially correlated to the internal quality, which mainly refers to the soluble solids content (SSC) and firmness [1,2].

Traditional investigation on SSC mainly depends on chemical analytical methods, and firmness is usually measured by the M-T puncture test [3], However, these methods are destructive, time-consuming and costly. Visible and near infrared spectroscopy (Vis/NIRS) is regarded as a fast and nondestructive technique, which have been used to predict the SSC and firmness of kiwifruit [4,5]. However, the Vis/NIRS technique has limitations because it only acquires the spectral data from a single point or from a small portion of the samples without providing spatially resolved information. Fruit such as kiwifruit are heterogeneous in composition and structure. The compositional and structural variations mean that Vis/NIRS measurement is unable to provide accurate or global information for kiwifruit. Dielectric spectroscopy is another nondestructive technique without the limitations above. It has been used to predict the SSC and firmness of a whole kiwifruit, but its applicability in kiwifruit needs to be improved [6]. 

Aroma is a comprehensive attribute of the ripeness of fruit. The aroma-related volatiles from fruit vary with the ripening time, and different varieties of fruit usually have different aromas at the optimal eating ripe stage. Previous research on kiwifruit showed that the characteristic volatiles were continuously changing in the softening period through to the full ripening period, and the difference was confirmed from kiwifruit at different ripening stages [7,8,9]. The changed volatiles include aldehydes, esters and alcohols, which were identified by the means of gas chromatography–mass spectrometry (GC-MS) [10]. These findings provided a potential strategy to predict the ripeness of kiwifruit by sensing its aroma volatiles [11]. Frank et al. [12] determined the relationship between volatile chemicals and flavor characteristics of “Hayward” kiwifruit by gas chromatography/mass spectrometry-olfactometry (GC/MS-O), but correlations have not been quantitatively defined. Moreover, sensory evaluation by humans is inevitably associated with problems such as individual preference, stability and reproducibility.

Electronic nose (E-nose) is an olfactory simulation of biological functions to identify simple or complex odors [13]. A typical E-nose system consists of a sensor array, a signal processing subsystem and a pattern recognition subsystem. When the volatiles from samples flow through the sensor array, the response of the sensors will be recorded as the “fingerprints” of the data. Then, signal processing and pattern recognition techniques are employed to analyze these fingerprints, thereby identifying the odors. E-nose technology provides a rapid and nondestructive alternative to sense the aroma volatiles, and this has been recognized as one of the best strategies to monitor the ripening process of fruit [14,15,16]. As the most crucial part, the sensor array has been made up of different kinds of gas sensors. Various studies have applied conductive polymer (CP) sensors [17,18], surface acoustic wave (SAW) sensors [19,20] and quartz crystal microbalance (QCM) sensors [21,22] in the E-nose system to detect the ripeness of fruit. Compared with these sensors, metal oxide semiconductor (MOS) sensors, which have the advantages of rapid response, high sensitivity and most importantly low cost, have also been widely used in E-nose applications [23]. 

Liu et al. [24] used a MOS E-nose system to detect the quality of kiwifruit. They mainly focused on the qualitative discrimination of kiwifruit at different storage times, but no other research has been reported for the further quantitative prediction of the internal quality of kiwifruit by E-nose. So, the relationship between E-nose data and the internal quality of kiwifruit has still not been identified in previous research. In this study, an E-nose with 10 MOS sensors was used to predict the ripeness of postharvest kiwifruit with different feature extraction methods and different pattern recognition methods. The main objectives of this study are: (1) to determine SSC, firmness and overall ripeness of postharvest kiwifruit during the ripening process; (2) to discriminate kiwifruit at different ripening times by the E-nose data based on different feature extraction methods; and (3) to predict overall ripeness, SSC and firmness by the E-nose data based on different pattern recognition algorithms.

## 2. Materials and Methods

### 2.1. Kiwifruit Samples

More than 200 kiwifruits (*Actinidia chinensis.* cv. Hongyang) at commercial maturity on September 25, 2017, were hand-picked from a local orchard in Shaoxing, China. Upon arrival at the laboratory in Zhejiang University, Hangzhou, they were weighed and selected. After removing the deformed and bruised ones, 160 kiwifruits (73.7 ± 6.1 g) remained as the samples. They were randomly divided into 8 groups, and each group contained 20 samples labeled from 1 to 20. These kiwifruits were stored in an incubator (STIK (Shanghai) CO., Shanghai, China) for postharvest ripening at the controlled natural condition of 20 °C and 70% relative humidity. According to the pre-experiment, about one week of storage in natural conditions should make the postharvest kiwifruit fully-ripe and edible. The first day, when the samples were picked and placed into the incubator was defined as day 0. One group of samples was taken out from the incubator and analyzed daily, and the experiment lasted for 8 days.

### 2.2. Electronic Nose Detection

In this study, a PEN3 E-nose system (Airsense Analytics GmBH, Schwerin, Germany) was employed to detect the sample aroma. The E-nose system consists of a sampling device, a sensor array and the system software for data acquisition and recording. As the most crucial part, the sensor array is composed of 10 MOS sensors, and each of them is sensitive to specific volatile compounds. Table 1 lists the 10 MOS sensors and describes their main applications and detection limitations.

**Figure 1:**
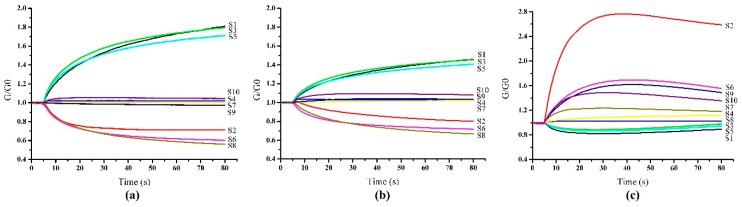
Typical response signals of E-nose system during the ripening process: (**a**) day 0; (**b**) day 4; (**c**) day 7.

The E-nose system was preheated for 1 h to reach a working temperature (above 200 °C) before detection and was cleaned by reference gas (clean air) after detection. Each kiwifruit was put into a glass beaker (500 mL) sealed with plastic cap for 1 h to ensure the volatile compounds which released from the kiwifruit to fill the beaker and to get equilibrium. To reduce the drift in MOS sensors, it was suggested that each sampling include an 80 s measurement process to get the stable signals and a 70 s cleaning process to normalize the sensors according to the preliminary experiment. The sample gas was pumped into the sensor chamber at a flow rate of 200 mL/min, and the signals per second were collected. All the E-nose detections were carried out at a temperature of 20 °C ± 0.5 °C and relative humidity of 70% ± 5 %. The typical response signals of E-nose system detecting from postharvest kiwifruit during the ripening period are shown in Figure 1, where the X-axis represents the time and the Y-axis represents the sensor signals. The signals were expressed by G/G0, where G and G0 represented the conductivities of sensors in the sample gas and in the clean gas, respectively.

As shown in Figure 1a–c, the results demonstrated that S1, S2, S3, S5, S6 and S8 were relatively sensitive to the sample gas during the ripening period. The signals of the array (S1, S3 and S5) decreased from day 0 to day 7, as a result of the decrease in aromatic volatiles. However, the signals of the array (S2, S6 and S8) increased with the increment in ripening days, which may be caused by the increase in methane and alcohol [10]. It should be noted that the response of S2 was the most significant. The signals of S2 always increased to the maximum very rapidly and then remained stable at a relatively high level, but the maximum magnitude was significantly different between early and late ripening days. The results indicated that the E-nose system responded sensitively to the change in aroma volatiles from postharvest kiwifruit during the ripening period. There was a potential relationship between the signals of E-nose and the ripeness of kiwifruit. 

### 2.3. Determination of SSC, Firmness and Overall Ripeness

The physical and chemical contents of postharvest kiwifruit change continuously during the ripening process [25]. In this study, two principal ripeness indexes of SSC and firmness were used to evaluate the sweetness and hardness of kiwifruit. 

The firmness of kiwifruit was measured by M-T puncture test using the Universal Testing Machine (Instron 5543, Instron Corp., Norwood, MA, USA). A cylindrical probe with a diameter of 6 mm was inserted into the samples with a penetration depth of 8 mm at a speed of 20 mm/min. Each sample was punctured at three sites with 120° intervals along the equatorial plane, and the force-distance (F-D) curve was acquired. Maximum force (the maximum value of each F-D curve) was determined as the firmness, which was used to evaluate the hardness of fruit [26]. The values at three sites were averaged to represent the global firmness for each sample. 

The whole kiwifruit was squeezed into juice by a blender (DESIGNER 675, Blendtec, Orem, UT, USA) after the puncture test, and the juice was filtered through 4 layers of 120 mesh cotton in order to remove the solid particles. The SSC of juice was measured by a digital refractometer (PR-101α, Atago Co., Ltd., Tokyo, Japan) to represent the sweetness for each sample. 

The sweetness and hardness, which were considered as the most important sensory factors, were combined to evaluate the overall ripeness of kiwifruit. The sweetness was rated using a scale of 1–5 based on SSC, and the hardness was also rated using a 1–5 scale based on firmness. The overall ripeness was classified into three different ripening stages (unripe, mid-ripe and eating ripe) based on the total scale of SSC and firmness. The postharvest kiwifruit at eating ripe stage had the optimal overall ripeness when the sweetness and hardness reached the best eating condition. The evaluation criteria for overall ripeness are described as shown in Table 2. 

### 2.4. Statistical Analysis of E-Nose Data

#### 2.4.1. Different Feature Extraction Methods

Previous research has revealed that different feature extraction methods for E-nose data might lead to different classification and prediction results [27,28]. According to the analysis of E-nose response signals above, three different feature extraction methods were tested to discriminate the kiwifruit at different ripening times in this study. They were: (1) the max/min values, the maximum or the minimum values of each response curve; (2) the difference values, the difference between the maximum and minimum values of each response curve; and (3) the 70th s values, the 70th s values of each response curve. Linear discriminant analysis (LDA) is a supervised algorithm to classify different objects by Fisher’s linear discriminant based on linear or quadratic combination of labeled data [29]. Here, LDA was applied to visualize the discrimination performances based on the above three feature extraction methods. 

#### 2.4.2. Quantitative Regression Methods

The pattern recognition algorithms of partial least squares regression (PLSR), support vector machine (SVM) and random forests (RF) were applied to build robust prediction models. PLSR is a commonly used regression method which combines the advantages of principle component analysis (PCA) and multiple linear regression (MLR). It is usually used to build high-performance models when there are high linear correlations among the variables. SVM is a supervised learning algorithm, which performs well on problems with small samples of non-linear and high-dimensional data, just like the E-nose data in this study [30]. To obtain the best performance, the penalty parameter C and the kernel parameter γ should be optimized. RF is an ensemble learning method with a combination of tree predictors based on voting theory. Each tree predictor acts as a classifier to vote for one class and the final output is the class with the most votes in the forest [31]. Recent studies have proved its potential in analyzing the E-nose data for nuts [27] and fruit [32]. 

#### 2.4.3. Distribution of Data Sets and Assessment of Models

In this study, 20 replicates were conducted for each group, and there was a total of 160 samples for the E-nose data set. In the data modeling, the data set was divided into two subsets including the training set (120 samples) and the testing set (40 samples). The results of discrimination were visualized by the LDA plot, and the performance was evaluated by the discrimination accuracy rate. For quantitative prediction, the performances of PLSR, SVM and RF were evaluated by two parameters: square correlation coefficient (R^2^) and root mean square error (RMSE). The larger R^2^ and the lower RMSE indicated the better prediction performance. 

The data processing method of LDA was performed by Statistical Product and Service Solutions v22.0 (International Business Machines Corporation, Armonk, USA). The PLSR method was performed by Minitab 17 (Minitab Inc., State College, PA, USA). LIBSVM [33] and the RF algorithms were run in MATLAB 2014b software (MathWorks, Natick, MA, USA). 

## 3. Results

### 3.1. Results of SSC, Firmness and Overall Ripeness Determination

SSC and firmness are two of the most important ripeness indexes for kiwifruit. Hence, these two indexes were measured daily after E-nose detection during the 8-day postharvest ripening period. Then the overall ripeness of kiwifruit was determined based on the evaluation criteria. The results of SSC, firmness and overall ripeness determination are presented in Table 3 and Table 4.

Table 3 displays the average values (±standard deviation) of SSC and firmness with the change in ripening day. The results showed that SSC increased rapidly in the first five days, and then rose gradually to an optimal eating condition of 14%–18% [2]. It could be seen that the firmness had no obvious difference in the first four days, but sharply declined from day 4 to day 6. At the final ripening stage, the firmness declined to an optimal eating condition of 1–5 N [10]. In the ripening process, the postharvest kiwifruit became increasingly sweet and soft until it obtained an optimal eating ripe condition with favorable SSC and firmness. 

Table 4 displays the quantity of samples, average values (±standard deviation) of SSC and firmness with the change in ripening stage. Based on the evaluation criteria for overall ripeness, 79 and 41 samples of the kiwifruit were separately evaluated at the unripe and mid-ripe stage, and the rest of the 40 samples were at the eating ripe condition. An increase in SSC and a decline in firmness was easily recognized with the increment in overall ripeness. Average SSC and firmness of postharvest kiwifruit were 16.48% and 4.44 N, respectively, at the eating ripe stage. These kiwifruit had the optimal eating taste with regard to sweetness and hardness. 

### 3.2. Discrimination of Different Ripening Times of Kiwifruit Based on LDA

**Figure 2:**
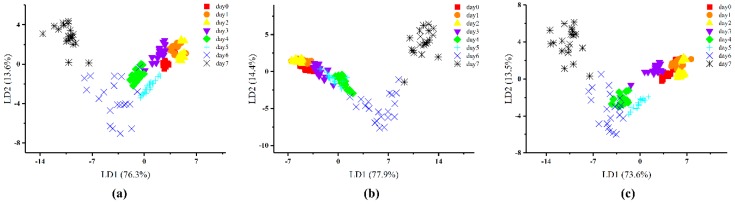
Linear discriminant analysis (LDA) by ripening day based on three different feature extraction methods: (**a**) the max/min values; (**b**) the difference values; (**c**) the 70th s values.

**Figure 3:**
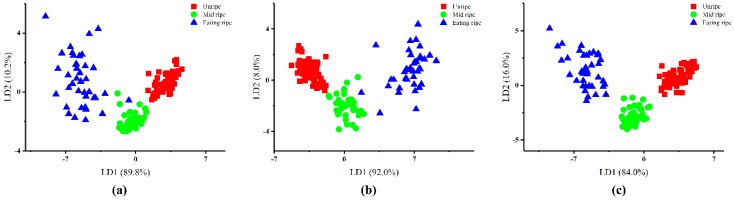
LDA by overall ripeness based on three different feature extraction methods: (**a**) the max/min values; (**b**) the difference values; (**c**) the 70th s values.

The leave-one-out method and cross-validation procedures were fused into LDA to analyze the E-nose data. A stepwise procedure was employed (variable to be included if F < 0.05, and variable to be removed if F > 0.10) for LDA variable selection, and Wilks’ Lambda test was carried out to confirm which discriminant function was significant. The distribution of samples at different ripening times was visualized by LDA plots based on the three different feature extraction methods as shown in Figure 2 and Figure 3. 

The results of LDA to discriminate the kiwifruit at different ripening days are presented in Figure 2. This demonstrates that the samples could be discriminated overall but there were still some overlaps. Figure 2a shows that the sum of LD1 and LD2 explains 89.9% of the total variance (LD1 = 76.3% and LD2 = 13.6%) by the max/min values method. Most of the samples could be discriminated except for some individuals. Figure 2b shows that the first two discriminant functions explain 92.3% of the total variance of the E-nose data (LD1 = 77.9% and LD2 = 14.4%) by the difference values method. More points overlapped in the LDA plot especially among the samples before day 4. The samples from day 0 today 4 could hardly be discriminated by the difference values method. As shown in Figure 2c, 87.1% of the total variance could be explained by LD1 and LD2 based on the 70th s values method (LD1 = 73.6% and LD2 = 13.5%). It can be seen that the within-group distances of samples are shortened but overlaps can be still be observed between samples day 0, day 1 and day 2, as well as between day 4 and day 6. According to Figure 2, the E-nose signals responded differently to the aroma volatiles from postharvest kiwifruit at different ripening days, however, the differences could not be totally discriminated, especially at the early days of the ripening process. Compared with the feature extraction methods of max/min and difference values, the 70th s values method performed best in discriminating the different ripening days of the postharvest kiwifruit.

The results of LDA to discriminate the kiwifruit with different overall ripeness are presented in Figure 3. The samples of postharvest kiwifruit were easily classified into three groups based on the overall ripeness, unripe, mid-ripe and eating ripe. Figure 3 showed that the sum of LD1 and LD2 could explain 100% of the variance by using the feature extraction methods of max/min, difference and 70th s values. From Figure 3a,b, it was observed that all the samples at the unripe and mid-ripe stage could be clearly discriminated, but some points at the mid-ripe and eating ripe stage overlapped in the max/min and difference values methods. The best discrimination results were obtained by the 70th s values method whereby all of the postharvest kiwifruit could be accurately discriminated, as shown in Figure 3c. 

The discrimination accuracy rate was investigated to quantitatively evaluate the discrimination performances of the three different feature extraction methods. Results of the discrimination by LDA are presented in Table 5. When grouped by ripening day, 93.1% of the samples in the original groups were successfully discriminated by the feature extraction method of 70th s values. After leave-one-out cross-validation calculations, 91.3% of the samples were successfully discriminated. Results of discrimination by the max/min values method showed that 93.1% of the samples from the original groups and 89.4% of the samples from the cross-validation groups were successfully discriminated. By using the difference values method, the original accuracy rate and cross-validation accuracy rate were 90.0% and 86.9%, respectively. In contrast, it was obvious that the discrimination accuracy rates were improved when samples were grouped by overall ripeness. The original accuracy rates were 100%, 98.8% and 98.8%, and the cross-validation accuracy rates were 99.4%, 97.5% and 98.8% for the feature extraction methods of 70th s values, max/min values and difference values, respectively. From the perspective of the feature extraction method, the 70th s values method performed better than the max/min and difference values methods in discriminating samples by ripening day and by overall ripeness. The best results were obtained in discriminating samples with different overall ripeness by the 70th s values method where the original accuracy rate and cross-validation accuracy rate reached 100% and 99.4%, respectively. Therefore, samples were grouped by overall ripeness, and 70th s values were extracted as the features for further prediction analysis in this study.

### 3.3. Quantitative Prediction of Overall Ripeness, SSC and Firmness

#### 3.3.1. Regression Results Based on PLSR

**Figure 4:**
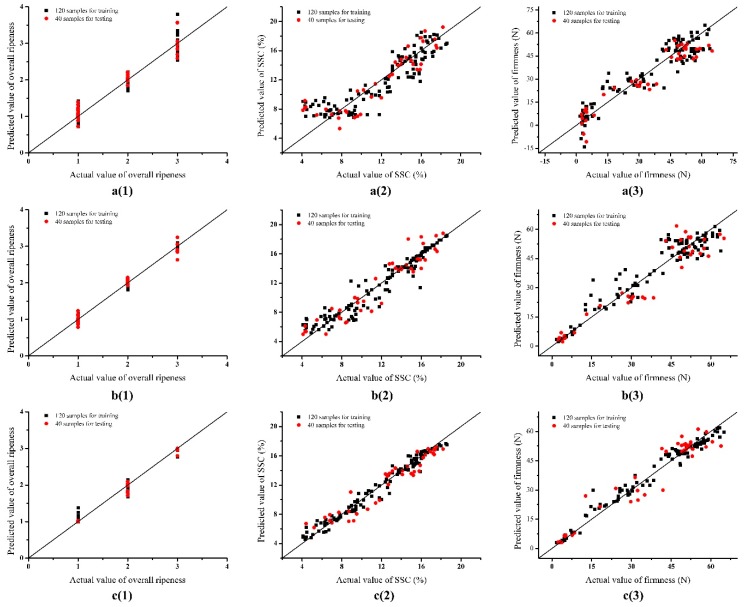
Predicted versus actual values from different prediction models: (**a**) presents PLSR, (**b**) presents SVM, and (**c**) presents RF; (1) stands for overall ripeness, (2) stands for SSC, and (3) stands for firmness.

PLSR is a method for multivariate statistical analysis that is suitable to solve the problems of prediction. Here, it was used to establish the correlations between E-nose data and ripeness indexes (overall ripeness, SSC and firmness). Figure 4a(1)–a(3) visualize the linear relationships between the predicted and actual values of overall ripeness, SSC and firmness, based on PLSR. We can see that overall ripeness, except for eating ripeness was well predicted. For SSC and firmness, the overall results were acceptable, but high deviations were observed in the low-level ranges. The evaluating parameters of R^2^ and RMSE in training and testing sets are listed in Table 6. The results showed a good correlation between E-nose data and overall ripeness (R^2^ = 0.9341 in the training set, R^2^ = 0.9430 in the testing set) but the performance of the PLSR model was unsatisfying in predicting SSC (R^2^ was only about 0.8 in training and testing sets) and firmness (R^2^ = 0.8848 in the training set, R^2^ = 0.9014 in the testing set). The results indicated that the E-nose data had better correlations with overall ripeness than with SSC and firmness. The performance of the prediction model had to be improved, especially in predicting SSC and firmness.

#### 3.3.2. Regression Results based on SVM

**Figure 5:**
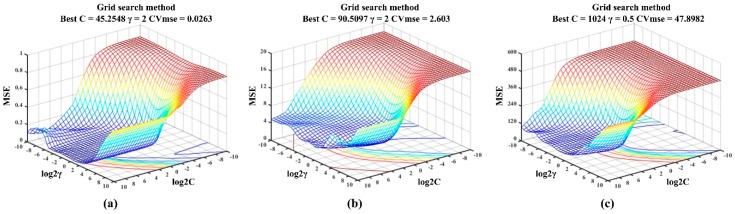
Searching of the penalty and kernel parameters for building the LIBSVM model: (**a**) overall ripeness; (**b**) SSC; (**c**) firmness.

In the approach of LIBSVM, radial basis function (RBF) was chosen as the core function and 5-fold cross-validation was applied. As LIBSVM is sensitive to the selection of penalty parameter C and kernel parameter γ, a grid search method was used to seek the best combination of C and γ with exponentially growing sequences of C and γ. Here, log2C and log2γ ranged from [−10, 10] at an interval of 0.5. Each combination of C and γ was checked by 5-fold cross-validation until the best cross-validation MSE (CVmse) was obtained. The search for grid points (log2C, log2γ) for the LIBSVM models is presented in Figure 5.

For overall ripeness, Figure 5a shows that CVmse reached the best of 0.0263 when the combination of C = 45.2548 and γ = 2 was searched. As shown in Figure 5b,c, the optimal combinations were searched to build the prediction models for SSC (C = 90.5097 and γ = 2) and firmness (C = 1024 and γ = 0.5). With these best combinations, the linear relationships between the predicted and actual values of ripeness indexes based on LIBSVM are visualized in Figure 4b(1)–b(3), and the regression results based on LIBSVM are presented in Table 6. The figures show that the performance of the prediction model based on LIBSVM was improved. Especially, a great improvement was achieved in predicting the eating ripeness and the low-level SSC and firmness. Results of the regression in Table 6, confirmed the high correlation between E-nose data and overall ripeness (R^2^ = 0.9921 in the training set, R^2^ = 0.9790 in the testing set). Furthermore, the values of R^2^ showed a noticeable improvement in predicting SSC (R^2^ = 0.9235 in the training set, R^2^ = 0.8948 in the testing set) and firmness (R^2^ = 0.9390 in the training set, R^2^ = 0.9128 in the testing set) based on LIBSVM.

#### 3.3.3. Regression Results Based on RF

**Figure 6:**
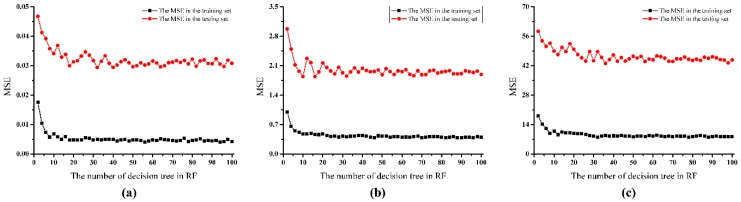
Searching of the decision trees for building the RF model: (**a**) overall ripeness; (**b**) SSC; (**c**) firmness.

RF is an ensemble of unpruned classification and regression trees (CART), and the trees are split to nodes by random subsets of variables. So, the main variables for the RF model are the number of decision trees (*n*_tree_) and the number of features (*m*_try_) in each tree. The default *m*_try_ value is the square root of the total number of sensors, and the value here is 3 for the E-nose system. A performance experiment was conducted based on the number of decision trees from 2 to 100 at a 2 trees interval, and MSEs in training and testing sets were considered as evaluation parameters. The results of searching decision trees for the RF model are presented in Figure 6. 

For overall ripeness, as shown in Figure 6a, MSEs in training and testing sets were relatively high when *n*_tree_ was smaller than 10. Then, the values fluctuated during the range of 10–40 for *n*_tree_. After *n*_tree_ exceeded 40, the MSEs remained in a stable low-level condition of 0.03 in the training set and 0.005 in the testing set. A similar tendency could be observed in searching decision trees for the RF models for SSC and firmness as shown in Figure 6b,c. Taking the prediction performance and computation time into consideration, the value of decision trees *n*_tree_ was determined as 40 for modeling the RF network. The linear relationships between the predicted and actual values of ripeness indexes based on RF are visualized in Figure 4c(1)–c(3), and the regression results based on RF are presented in Table 6. More predicted values were located near the actual values, and deviations were further reduced based on RF, as shown in Figure 4. Regression results based on RF were further improved in predicting all of the three ripeness indexes. Overall ripeness was perfectly predicted in the training set with R^2^ = 0.9928 and in the testing set with R^2^ = 0.9928. Large values of R^2^ were also obtained in predicting SSC and firmness (R^2^ > 0.97 in the training set, R^2^ > 0.91 in the testing set). It could be seen that the prediction model based on RF had the best performance compared with the models based on PLSR and SVM. 

## 4. Discussion

This study explored the potential to predict the ripeness of postharvest kiwifruit using a MOS E-nose. Different feature extraction methods and different pattern recognition methods were combined to reach this goal. The results showed that the MOS E-nose could effectively discriminate the samples at different ripening stages and predict the overall ripeness, SSC and firmness. 

An increase in SSC and a decrease in firmness could be observed as the number of ripening days increased. The changing pattern for SSC and firmness could be described as having three phases: a slow initial phase followed by a fast phase, and then, a final slow phase, which was similarly obtained by Burdon, et al. [34]. The fast phase usually lasts for four days and is associated with respiratory climacteric and ethylene production [35]. The duration of the initial and final slow phases depend on the harvest date and storage time. In this study, the first slow phase lasted for four days because the kiwifruit were harvested at an early maturity date. The ripeness only had a minor increase prior to harvest on the vine, which resulted in a longer first phase. 

Results of LDA discrimination of kiwifruit at different ripening times showed that discrimination performance was improved when grouping samples by overall ripeness. The reason for this may be that the volatile compounds of postharvest kiwifruit were relatively stable in the initial days. After this period, more volatile compounds began to volatilize from the kiwifruit with the decline of firmness. So, the postharvest kiwifruit had distinguished aromas at different ripening stages, and these differences could be easily discriminated by overall ripeness, not by ripening day. The research conducted by Yi et al. [25] revealed by the use of GC-MS that ester and aldehyde headspace compounds were very different in the inedible and edible stages in the Hayward kiwifruit. This characteristic is an advantage in discriminating the optimal eating ripeness for postharvest kiwifruit by E-nose technology. Grouping fruit by overall ripeness was more reliable, and this method has also been adopted in studies on the banana [16], mango [17] and peach [36]. Previously, Liu et al. [24] used a MOS E-nose system to detect the quality of kiwifruit and classified the samples at different storage days. Seven groups with 5 replicates were poorly classified, which is similar to the results presented in Figure 2. 

The feature extraction method of 70th s values performed better than the other two methods in discriminating kiwifruit samples. It seems the 70th s values may contain more combined information for the E-nose data, which are usually extracted as the features in previous research [27,28]. However, the 70th s values had limitations in providing comprehensive information for E-nose data analysis. With the development of feature mining technology, new methods like genetic algorithms, evolutionary algorithms [37] and features fusion strategies [38] have been introduced for solving feature selection problems in E-nose applications. This is one of the most important research areas for E-nose applications in the future.

Quantitative predictions of the internal quality of kiwifruit by E-nose have not been reported before. In this study, overall ripeness, SSC and firmness were predicted by E-nose combined with different pattern recognition methods. Results demonstrated that the RF model showed an advantage in predicting overall ripeness, SSC and firmness, which means that the RF algorithm was able to extract useful information more effectively than PLSR and SVM in processing the E-nose data collected for kiwifruit volatiles. The advanced performance of the RF algorithm in applications of E-nose detection was also confirmed by Liu et al. [39] and Qiu et al. [40]. Compared with the previous studies by Vis/NIR spectroscopy [4] and dielectric spectroscopy [6], the prediction of both SSC and firmness was improved by E-nose combined with RF. 

The results of this study revealed the high correlations between E-nose signals and the internal quality of kiwifruit. It suggested that the E-nose technique combined with chemometrics, could be a new approach to predict the ripeness of postharvest kiwifruit. However, specific aroma volatiles of kiwifruit during ripening that may have significant influence on E-nose responses were still not identified. Future work could focus on the determination of these aroma volatiles by means of GC-MS. Based on this, more reliable E-nose systems will be developed according to the selection of highly sensitive gas sensors. Moreover, E-nose can be applied to detect the quality of kiwifruit during transportation and storage. 

## 5. Conclusions

This study attempted to predict the ripeness of postharvest kiwifruit during ripening by a MOS electronic nose combined with chemometrics. After processing and analyzing the data based on different feature extraction methods and different pattern recognition methods, the following conclusions are drawn:

1. The overall ripeness of postharvest kiwifruit was classified into three ripening stages (unripe, mid-ripe and eating ripe) based on the evaluation criteria. The average SSC and firmness of postharvest kiwifruit was 16.48% and 4.44 N, respectively, at the eating ripe stage.
2. The LDA results based on three different feature extraction methods showed that the samples at different ripening times could be discriminated. The 70th s values method had the best performance in discriminating the samples with different overall ripeness with an original accuracy rate of 100% and a 99.4% cross-validation accuracy rate.
3. The regression results based on different pattern recognition methods showed that the overall ripeness, SSC and firmness of postharvest kiwifruit could be well predicted. The RF algorithm had the best performance in predicting the three ripeness indexes with higher R^2^ and lower RMSE compared with PLSR and SVM.

According to the above conclusions, the ripeness of postharvest kiwifruit was successfully predicted by the MOS E-nose system combined with chemometrics. Specifically, overall ripeness, SSC and firmness were well predicted by the E-nose date based on pattern recognition algorithms. The results illustrated that E-nose signals had high correlations with ripeness characteristics of postharvest kiwifruit. This study proved that aroma volatiles are comprehensive attributes for the ripeness of kiwifruit, which indicated that the E-nose technique could be an accurate and comprehensive approach to predict the ripeness of kiwifruit. 

## Figures and Tables

**Table 1:** Sensors in PEN3 E-nose system and their sensitivity description.

Number	Name	Sensitive Substances	Reference
S1	W1C	Aromatic compounds	Toluene, 10 ppm
S2	W5S	Very sensitive, broad range sensitivity, react on nitrogen oxides, very sensitive with negative signal	NO_2_, 1 ppm
S3	W3C	Ammonia, used as sensor for aromatic compounds	Propane, 1 ppm
S4	W6S	Mainly hydrogen, selectively, (breath gases)	H_2_, 100 ppb
S5	W5C	Alkanes, aromatic compounds, less polar compounds	Propane, 1 ppm
S6	W1S	Sensitive to methane (environment) ca. 10 ppm. Broad range, similar to No. 8	CH_3_, 100 ppm
S7	W1W	Reacts on sulphur compounds, H_2_S 0.1 ppm. Otherwise sensitive to many terpenes and sulphur organic compounds, which are important for smell, limonene, pyrazine	H_2_S, 1 ppm
S8	W2S	Detects alcohol’s, partially aromatic compounds, broad range	CO, 100 ppm
S9	W2W	Aromatics compounds, sulphur organic compounds	H_2_S, 1 ppm
S10	W3S	Reacts on high concentrations >100 ppm, sometime very selective (methane)	CH_3_, 10CH_3_, 100 ppm

**Table 2:** The evaluation criteria for overall ripeness.

**Scale**	**1**	**2**		**3**	**4**		**5**
SSC (%)	<6	6–10		10–14	14–18		>18
Firmness (N)	>15	10–15		5–10	1–5		<1
**Total scale**	**2–5**		**6–8**			**9–10**	
Overall ripeness	Unripe		Mid ripe			Eating ripe	

**Table 3:** Average values (±standard deviation) of soluble solids content (SSC) and firmness at different ripening days.

Ripening Day	day0	day1	day2	day3	day4	day5	day6	day7
SSC (%)	5.12 (±0.97)	7.51 (±1.18)	8.92 (±1.26)	11.36 (±1.23)	14.12 (±0.66)	14.46 (±1.32)	16.13 (±1.03)	16.82 (±1.14)
Firmness (N)	50.62 (±4.18)	53.06 (±5.38)	54.03 (±7.02)	51.29 (±6.24)	31.06 (±5.38)	21.76 (±6.46)	5.77 (±1.90)	3.12 (±0.65)

**Table 4:** Quantity of samples, average values (±standard deviation) of SSC and firmness at different ripening stages.

Overall Ripeness	Unripe	Mid Ripe	Eating Ripe
Quantity	79	41	40
SSC (%)	8.16 (±2.50)	14.26 (±1.05)	16.48 (±1.13)
Firmness (N)	52.30 (±5.88)	26.95 (±8.20)	4.44 (±1.94)

**Table 5:** Results of the discrimination by LDA based on three different feature extraction methods.

Feature Extraction Methods	Ripening Day		Overall Ripeness	
	The Original Accuracy Rate (%)	The Cross-Validation Accuracy Rate (%)	The Original Accuracy Rate (%)	The Cross-Validation Accuracy Rate (%)
Max/Min values	93.1	89.4	98.8	97.5
Difference values	90.0	86.9	98.8	98.8
70th s values	93.1	91.3	100.0	99.4

**Table 6:** The results of evaluating parameters for prediction models based on PLSR, SVM and RF.

Algorithms	Ripeness Indexes	Training Set		Testing Set	
		R^2^	RMSE	R^2^	RMSE
PLSR	Overall ripeness	0.9341	0.2107	0.9430	0.2075
	SSC	0.7931	1.9649	0.8015	1.8969
	Firmness	0.8848	7.1668	0.9014	7.1583
SVM	Overall ripeness	0.9921	0.0770	0.9790	0.1205
	SSC	0.9235	1.1590	0.8948	1.4041
	Firmness	0.9390	5.1424	0.9128	6.3457
RF	Overall ripeness	0.9928	0.0684	0.9928	0.0722
	SSC	0.9749	0.6675	0.9143	1.1957
	Firmness	0.9814	2.9343	0.9290	5.3901

## References

[1] Crisosto C.H., Crisosto G.M. (2001). Understanding consumer acceptance of early harvested ‘Hayward’ kiwifruit. Postharvest Biol. Technol..

[2] Burdon J., McLeod D., Lallu N., Gamble J., Petley M., Gunson A. (2004). Consumer evaluation of “Hayward” kiwifruit of different at-harvest dry matter contents. Postharvest Biol. Technol..

[3] Gómez A.H., Wang J., Pereira A.G. (2005). Impulse response of pear fruit and its relation to Magness-Taylor firmness during storage. Postharvest Biol. Technol..

[4] Moghimi A., Aghkhani M.H., Sazgarnia A., Sarmad M. (2010). Vis/NIR spectroscopy and chemometrics for the prediction of soluble solids content and acidity (pH) of kiwifruit. Biosys. Eng..

[5] Guo W.C., Zhao F., Dong J.L. (2016). Nondestructive measurement of soluble solids content of kiwifruits using near-infrared hyperspectral imaging. Food Anal. Meth..

[6] Ragni L., Cevoli C., Berardinelli A., Silaghi F.A. (2012). Non-destructive internal quality assessment of “Hayward” kiwifruit by waveguide spectroscopy. J. Food Eng..

[7] Young H., Paterson V.J. (1985). The effects of harvest maturity, ripeness and storage on kiwifruit aroma. J. Sci. Food Agric..

[8] Friel E.N., Wang M., Taylor A.J., MacRae E.A. (2007). In vitro and in vivo release of aroma compounds from yellow-fleshed kiwifruit. J. Agric. Food Chem..

[9] Garcia C.V., Stevenson R.J., Atkinson R.G., Winz R.A., Quek S.Y. (2013). Changes in the bound aroma profiles of ‘Hayward’ and ‘Hort16A’ kiwifruit (*Actinidia* spp.) during ripening and GC-olfactometry analysis. Food Chem..

[10] Wang M.Y., MacRae E., Wohlers M., Marsh K. (2011). Changes in volatile production and sensory quality of kiwifruit during fruit maturation in Actinidia deliciosa ‘Hayward’ and A. chinensis ‘Hort16A’. Postharvest Biol. Technol..

[11] Baietto M., Wilson A.D. (2015). Electronic-nose applications for fruit identification, ripeness and quality grading. Sensors.

[12] Frank D., O’Riordan P., Varelis P., Zabaras D., Watkins P., Ceccato C., Wijesundera C. (2007). Deconstruction and recreation of ‘Hayward’ volatile flavour using a trained sensory panel, olfactometry and a kiwifruit model matrix. Acta Hortic..

[13] Gardner J.W., Bartlett P.N. (1994). A brief-history of electronic noses. Sens. Actuator B Chem..

[14] Sanaeifar A., Mohtasebi S.S., Ghasemi-Varnamkhasti M., Ahmadi H. (2016). Application of MOS based electronic nose for the prediction of banana quality properties. Measurement.

[15] Xu S., Lü E., Lu H., Zhou Z., Wang Y., Yang J., Wang Y. (2016). Quality detection of litchi stored in different environments using an electronic nose. Sensors.

[16] Chen L.Y., Wu C.C., Chou T.I., Chiu S.W., Tang K.T. (2018). Development of a dual MOS Electronic nose/camera system for improving fruit ripeness classification. Sensors.

[17] Zakaria A., Shakaff A.Y.M., Masnan M.J., Saad F.S.A., Adom A.H., Ahmad M.N., Jaafar M.N., Abdullah A., Kamarudin L.M. (2012). Improved maturity and ripeness classifications of *Magnifera Indica* cv. Harumanis mangoes through sensor fusion of an electronic nose and acoustic sensor. Sensors.

[18] Pathange L.P., Mallikarjunan P., Marini R.P., O‘Keefe S., Vaughan D. (2006). Non-destructive evaluation of apple maturity using an electronic nose system. J. Food Eng..

[19] Du X., Olmstead J., Rouseff R. (2012). Comparison of fast gas chomatography-surface acoustic wave (FGC-SAW) detection and GC-MS for characterizing blueberry cultivars and maturity. J. Agric. Food Chem..

[20] Hasanuddin N.H., Wahid M.H.A., Shahimin M.M., Hambali N.A.M.A., Yusof N.R., Nazir N.S., Khairuddin N.Z., Azidin M.A.M. Metal oxide based surface acoustic wave sensors for fruits maturity detection. Proceedings of the 2016 3rd International Conference on Electronic Design (ICED).

[21] De Lerma N.L., Moreno J., Peinado R.A. (2014). Determination of the optimum sun-drying time for *Vitis vinifera* L. cv. Tempranillo grapes by E-nose analysis and characterization of their volatile composition. Food Bioprocess Technol..

[22] Ali S.B., Ghatak B., Gupta S.D., Debabhuti N., Chakraborty P., Sharma P., Ghosh A., Tudu B., Mitra S., Sarkar M.P. (2016). Detection of 3-Carene in mango using a quartz crystal microbalance sensor. Sens. Actuator B Chem..

[23] Xu K.M., Wang J., Wei Z.B., Deng F.F., Wang Y.W., Cheng S.M. (2017). An optimization of the MOS electronic nose sensor array for the detection of Chinese pecan quality. J. Food Eng..

[24] Liu W., Hui G.H. (2015). Kiwi fruit (*Actinidia chinensis*) quality determination based on surface acoustic wave resonator combined with electronic nose. Bioengineered.

[25] Yi J.J., Kebede B.T., Grauwet T., Van Loey A., Hu X.S., Hendrickx M. (2016). A multivariate approach into physicochemical, biochemical and aromatic quality changes of puree based on Hayward kiwifruit during the final phase of ripening. Postharvest Biol. Technol..

[26] Li H., Pidakala P., Billing D., Burdon J. (2016). Kiwifruit firmness: Measurement by penetrometer and non-destructive devices. Postharvest Biol. Technol..

[27] Jiang S., Wang J. (2016). Internal quality detection of Chinese pecans (*Carya cathayensis*) during storage using electronic nose responses combined with physicochemical methods. Postharvest Biol. Technol..

[28] Wei Z., Wang J., Zhang W. (2015). Detecting internal quality of peanuts during storage using electronic nose responses combined with physicochemical methods. Food Chem..

[29] Safo S.E., Ahn J. (2016). General sparse multi-class linear discriminant analysis. Comput. Stat. Data Anal..

[30] Cortes C., Vapnik V. (1995). Support-vector networks. Mach. Learn..

[31] Breiman L. (2001). Random forests. Mach. Learn..

[32] Qiu S.S., Wang J. (2017). The prediction of food additives in the fruit juice based on electronic nose with chemometrics. Food Chem..

[33] Chang C.C., Lin C.J. (2011). LIBSVM: A library for support vector machines. ACM Trans. Intell. Syst. Technol..

[34] Burdon J., Lallu N., Pidakala P., Barnett A. (2013). soluble solids accumulation and postharvest performance of ‘Hayward’ kiwifruit. Postharvest Biol. Technol..

[35] Schroder R., Atkinson R.G. (2006). Kiwifruit cell walls: Towards an understanding of softening?. NZ J. Forest. Sci..

[36] Zhang H.M., Wang J., Ye S., Chang M.X. (2012). Application of electronic nose and statistical analysis to predict quality indices of peach. Food Bioprocess Technol..

[37] Jeong Y.S., Shin K.S., Jeong M.K. (2015). An evolutionary algorithm with the partial sequential forward floating search mutation for large-scale feature selection problems. J. Oper. Res. Soc..

[38] Men H., Shi Y., Jiao Y., Gong F., Liu J. (2018). Electronic nose sensors data feature mining: A synergetic strategy for the classification of beer. Anal. Methods.

[39] Liu M., Wang M., Wang J., Li D. (2013). Comparison of random forest, support vector machine and back propagation neural network for electronic tongue data classification: Application to the recognition of orange beverage and Chinese vinegar. Sens. Actuator B Chem..

[40] Qiu S., Wang J., Tang C., Du D. (2015). Comparison of ELM, RF, and SVM on E-nose and E-tongue to trace the quality status of mandarin (Citrus unshiu Marc.). J. Food Eng..

